# Reconfigurable control of coherence, dissipation, and nonreciprocity in cavity magnonics

**DOI:** 10.1038/s41598-025-15983-w

**Published:** 2025-08-22

**Authors:** Jintao Shuai, Bojong Kim, Junyoung Kim, Rutvij Bhavsar, Sang-Koog Kim

**Affiliations:** 1 National Creative Research Initiative Center for Spin Dynamics and Spin-Wave Devices, Nanospinics Laboratory, Department of Materials Science and Engineering, Seoul National University, Seoul, 08826 Republic of Korea; 2 School of Electrical Engineering, Korea Advanced Institute of Science and Technology (KAIST), Daejeon, 34141 Republic of Korea

**Keywords:** Applied physics, Magnetic properties and materials, Spintronics

## Abstract

Precise control of coupling strength, damping rate and nonreciprocity in photon–magnon systems is essential for advancing hybrid quantum technologies, including reconfigurable microwave components and quantum transducers. Here, we demonstrate magnetic field angle-dependent control of photon–magnon coupling and magnon dissipation in a cross-shaped microwave cavity supporting a spatially nonuniform radio-frequency (rf) magnetic field. By rotating the external magnetic field angle $\theta$ relative to the normal of the transmission line within the cavity plane, we simultaneously control the coherent coupling strength ${g}_{0}$, the ferromagnetic resonance (FMR) damping rate, and the system’s nonreciprocal response. The nonuniform rf field selectively excites both the uniform FMR mode and finite-wavevector spin waves in an Yttrium Iron Garnet (YIG) film, enabling angle-dependent two-magnon scattering. While typically regarded as a passive loss mechanism, we show that two-magnon scattering can serve as a dynamic and reversible knob to control magnon damping. Moreover, we realise nonreciprocity originating from the spatial asymmetry of the rf field, in addition to conventional phase-based schemes. These findings introduce new opportunities for in situ control of coherence, dissipation, and nonreciprocity in cavity magnonics, with broad implications for reconfigurable quantum and spintronic systems.

**Supplementary Information:**

The online version contains supplementary material available at 10.1038/s41598-025-15983-w.

## Introduction

Hybrid photon–magnon systems have garnered increasing attention as versatile platforms for exploring light-matter interactions and quantum technologies^1–4^. By coupling collective spin excitations (magnons) in ferrimagnetic materials such as Yttrium Iron Garnet (YIG) to microwave photons in a cavity, these systems exhibit strong light–matter interactions, enabling coherent signal processing, quantum state transfer, and nonreciprocal devices for isolators or circulators^5–14^. A major bottleneck in advancing cavity magnonic systems lies in the ability to dynamically and reversibly control key parameters: the photon–magnon coupling strength, the magnon damping rate, and the system’s nonreciprocal response^15–18^. While prior efforts have primarily focused on tuning the coherent coupling via positioning the YIG in the cavity or modifying structural design of the cavity, dynamically tuning magnon dissipation remains limited^15^. This is primarily because magnon damping is generally determined by intrinsic properties and fixed inhomogeneities introduced during fabrication, limiting real-time reconfiguration of device characteristics.

Magnon damping arises from both intrinsic and extrinsic processes. Intrinsic damping typically corresponds to Gilbert-like relaxation. Extrinsic damping encompasses additional decay channels induced by material imperfections, such as two-magnon scattering, which depend on inhomogeneities like defects or surface roughness^19,20^. In ferromagnetic resonance (FMR), two-magnon scattering involves coupling between the uniform mode and degenerate spin waves with finite wavevectors $\varvec{k}$, leading to additional dissipation channels. In cavity-based systems, the microwave field includes both standing and travelling wave components, with the latter contributing to radiative damping via photon-mediated channels^21^. To clarify terminology, we redefine “intrinsic” damping as dissipation processes that do not involve direct coupling to travelling photons (e.g., two-magnon scattering), while “extrinsic” damping refers to radiative coupling between magnons and travelling photons^13,15,16,18,22–24^. This distinction enables a more precise understanding of magnon damping in cavity-based systems and clarifies the independent roles of magnon–magnon and magnon–photon interactions.

Previous approaches to tuning magnon damping in photon–magnon hybrid systems have often relied on repositioning the YIG sample within a cavity or modifying the design of the cavity to adjust its coupling to electromagnetic modes^15^. These approaches influence dissipative photon–magnon interactions, however, it does not independently control over two-magnon scattering. Other techniques, including altering film deposition conditions and substrate structures, can modify magnon dissipation but are not suited for dynamic and in situ tuning^19,25–28^. The ability to dynamically modulate intrinsic damping — and to harness it as a functional degree of freedom — remains an unmet challenge in cavity magnonics.

Here, we address this challenge by introducing an angle-dependent control scheme based on a cross-shaped microwave cavity supporting a spatially nonuniform radio-frequency (rf) field. We demonstrate that simply rotating the in-plane magnetic field angle $\theta$ reconfigures the magnon excitation in YIG film, selectively activating finite-wavevector modes and thereby modulating two-magnon scattering. This configuration enables dynamic and reversible control over (i) the coherent photon–magnon coupling strength, (ii) the overall FMR damping rate (both intrinsic and extrinsic), and (iii) a pronounced nonreciprocal transmission behaviour. Importantly, the nonreciprocity observed in our system originates not only from the usual phase differences in forward- and backward-propagating waves^21^, but also from the spatial asymmetry of the rf magnetic field, which alters the FMR excitation conditions for forward and backward directions. This mechanism represents a novel route for directional control in cavity-based systems, with potential advantages in device design simplicity and reconfigurability. Our results establish two-magnon scattering as a tunable control parameter in cavity magnonics, not merely a passive dissipation channel. This opens a previously unexplored avenue for tuning coherence, dissipation, and directionality in hybrid magnonic systems using a geometrically simple and experimentally accessible setup. This conceptual advance points toward new directions in reconfigurable quantum interfaces, microwave nonreciprocal components, and spintronic signal processing.

## Results

### Experimental design

**Fig. 1:**
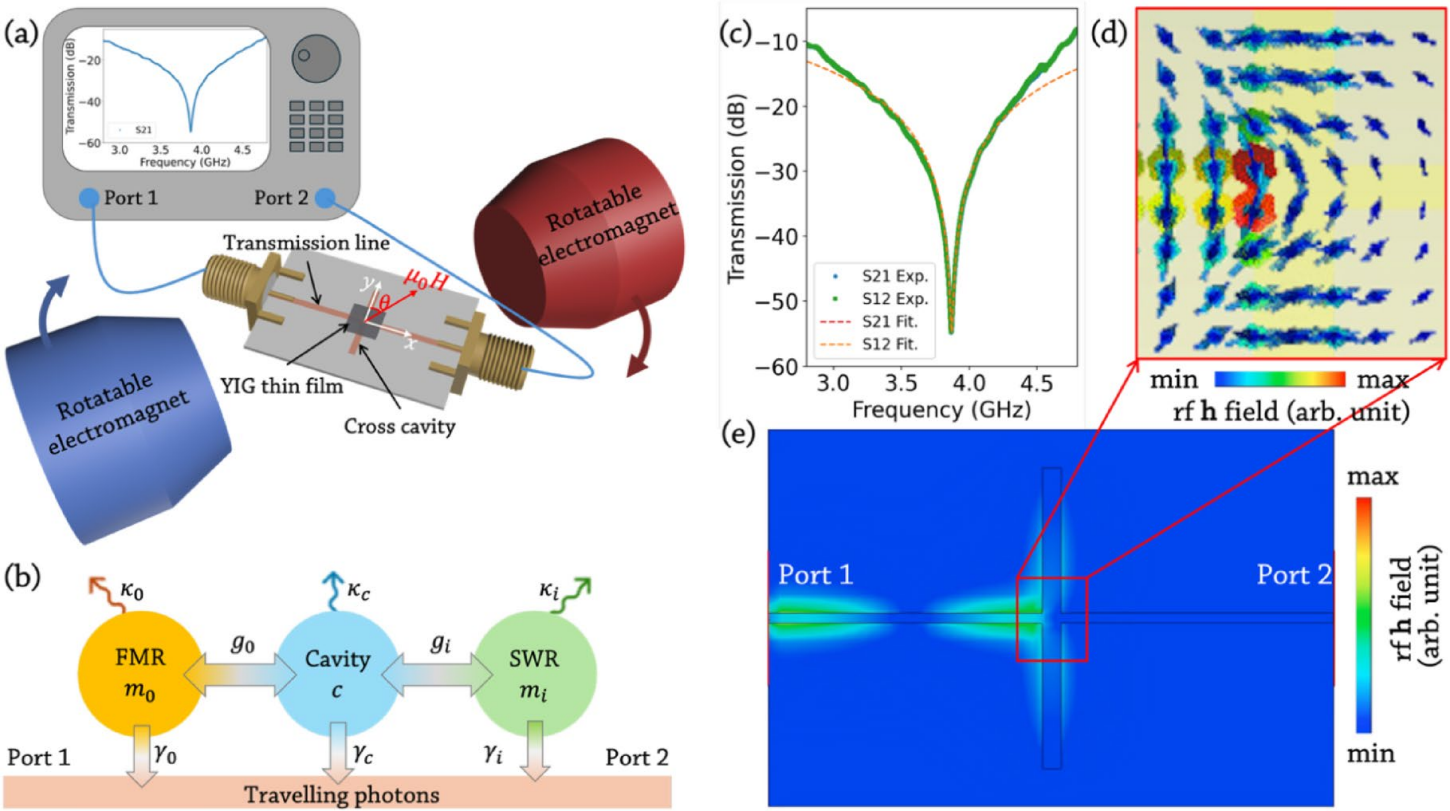
(**a**) Schematic of the experimental setup (not to scale). An YIG film is positioned at the centre of a cross-shaped cavity supporting both travelling and standing waves. A rotatable external magnetic field tunes the FMR frequency. The cavity’s two ports are connected to a VNA for measuring $\left|{\text{S}}_{21}\right|$ and $\left|{\text{S}}_{12}\right|$. (**b**) Diagram of the photon–magnon coupling mechanism. The cavity mode couples directly to both the FMR and SWR modes, with each mode dissipatively coupled to travelling photons at the input and output ports. (**c**) Measured $\left|{\text{S}}_{21}\right|$ and $\left|{\text{S}}_{12}\right|$ of the empty cavity, with fitting results overlaid. Lines are guides to the eye. (**d**) CST simulation of the $\mathbf{h}$ field orientation in the YIG region at the resonant frequency with rf signals entering from Port 1. (**e**) Heatmap of the simulated magnetic field (**h** field) intensity at resonance, with rf signals injected from Port 1. The red boxed area shows the region, drawn to scale, that is covered by the YIG thin film.

Figure 1(a) illustrates the experimental design. A YIG film is positioned at the cavity centre in a flip-chip configuration (defined as the origin). The cross-shaped microwave cavity comprises a central transmission line along the $x$-axis intersecting two orthogonal arms along the $y$-axis (Fig. 1(e)). This structure supports both travelling and standing waves. The YIG thin film and the cross-shaped cavity in this study only partial overlap (dimensions of the thin film and the cavity are detailed in Method section). In Fig. 1(e), a red box drawn to scale indicates the area covered by the YIG thin film. The orientation of the $\mathbf{h}$ field within the YIG film is depicted in Fig. 1(d). The nonuniformity of the $\mathbf{h}$ field in the cavity, especially the relative dimensions of the metallic structure and the YIG thin film, has been shown to play an important role in determining the coupling strength between the photon mode and the magnon mode^22^. An external magnetic field is applied to tune the magnon resonance frequency ${\omega}_{0}$. The magnetic field is applied with field direction $\theta$ from $0^\circ$ (perpendicular to the transmission line) to $90^\circ$ (parallel) within the $x$-$y$ plane. A calibrated vector network analyser (VNA) measures the transmission properties of the photon–magnon hybrid system, including $\left|{\text{S}}_{21}\right|$ (rf signals sent from Port 1 to Port 2) and $\left|{\text{S}}_{12}\right|$ (rf signals sent from Port 2 to Port 1). Figure 1(c) shows the measured $\left|{\text{S}}_{21}\right|$ and $\left|{\text{S}}_{12}\right|$ of the empty cavity, with a resonant frequency of ${\omega}_{c}/2\pi=3.85\:\text{G}\text{H}\text{z}$. The fitted intrinsic and extrinsic damping rates are ${\kappa}_{c}/2\pi=13.74\:\text{M}\text{H}\text{z}$ and ${\gamma}_{c}/2\pi=3.65\:\text{G}\text{H}\text{z}$, respectively, with the fitted curves overlaid. The near-identical $\left|{\text{S}}_{21}\right|$ and $\left|{\text{S}}_{12}\right|$ responses confirm the reciprocity of the cavity.

### Theoretical model

Figure 1(b) shows the diagram of the photon–magnon coupling mechanism in our work. Our system comprises a YIG film coupled to a microwave cavity. Under the excitation of a rf magnetic field $\mathbf{h}$, the fundamental FMR mode is driven, characterised by the in-phase precession of spins^29^. In addition to this uniform mode, spin wave resonance (SWR) modes with nonzero wavevector $\varvec{k}$ can also be excited, primarily due to the nonuniform rf field. These SWR modes involve collective excitations of a large spin population and can directly couple with the photon modes (shown in Fig. 3(c) and (e)). The Hamiltonian of the hybrid cavity-magnon system can be described as^29,30^:

1 $${\hat{H}}_{\text{s}\text{y}\text{s}}=\hslash{\omega}_{c}{c}^{\dag}c+\hslash\sum_{j=0}^{3}{\omega}_{{m}_{j}}{m}_{j}^{\dag}{m}_{j}+{\hat{H}}_{\text{i}\text{n}\text{t}}\mathrm{,}$$

where $c$ and ${c}^{\dag}$ are the annihilation and creation operators for the cavity mode. Similarly, ${m}_{0}$ and ${m}_{0}^{\dag}$ are the annihilation and creation operators for the FMR mode ($\:j=0$), while ${m}_{j}$ and ${m}_{j}^{\dag}$ represent the corresponding operators for the $j$^th^ (for *j* ∈ {1,2,3}) SWR mode.

Under the rotating wave approximation, the interaction Hamiltonian takes the form^29,30^:

$${\hat{H}}_{\text{i}\text{n}\text{t}}={\hslash}\left(\sum_{j=0}^{3}{g}_{j}\left(\theta\right)\left({c}^{\dag}{m}_{j}+{m}_{j}^{\dag}\text{c}\right)\right)\mathrm{,}$$

where $\theta$ is the angle of the external magnetic field relative to the $x$*-*axis, and ${g}_{j}\left(\theta\right)$ (for $j\in\{0,1,2,3\}$) denotes the coupling strength between the cavity as a function of the angle $\theta$ and the $j$^th^ mode.

We can then derive the quantum Langevin equation following the recipe in^21^ – i.e. the equations of motion for all the observables.

2 $$\:\begin{array}{c}{\partial}_{t}{\mathbf{x}}^{T}=-i\varOmega\:{\mathbf{x}}^{T}+{\mathbf{b}}^{T}{p}_{\text{i}\text{n}}\end{array}\mathrm{,}$$

$$\mathrm{where}\:\mathbf{x}=(c,{m}_{0},{m}_{1},{m}_{2},{m}_{3}),\:\mathbf{b}=(\sqrt{{\gamma}_{c}},\sqrt{{\gamma}_{0}}{e}^{i{\Theta}},\sqrt{{\gamma}_{1}}{e}^{i{\Theta}},\sqrt{{\gamma}_{2}}{e}^{i{\Theta}},\sqrt{{\gamma}_{3}}{e}^{i{\Theta}})\:\mathrm{and}$$

$$\:{\Omega\:}=\left(\begin{array}{cccc}{\omega}_{c}-i({\gamma}_{c}+{\kappa}_{c})&\:{g}_{0}\left(\theta\right)-i({e}^{i{\Theta}}\sqrt{{\gamma}_{0}{\gamma}_{c}}+\sqrt{{\kappa}_{c}{\kappa}_{0}})&\:{g}_{1}\left(\theta\right)-i({e}^{i{\Theta}}\sqrt{{\gamma}_{1}{\gamma}_{c}}+\sqrt{{\kappa}_{c}{\kappa}_{1}})&\:\cdots\:\\\:{g}_{0}\left(\theta\right)-i({e}^{i{\Theta}}\sqrt{{\gamma}_{c}{\gamma}_{0}}+\sqrt{{\kappa}_{0}{\kappa}_{c}})&\:{\omega}_{0}-i({\gamma}_{0}+{\kappa}_{0})&\:-i(\sqrt{{\gamma}_{1}{\gamma}_{0}}+\sqrt{{\kappa}_{1}{\kappa}_{0}})&\:\cdots\:\\\:{g}_{1}\left(\theta\right)-i({e}^{i{\Theta}}\sqrt{{\gamma}_{c}{\gamma}_{1}}+\sqrt{{\kappa}_{1}{\kappa}_{c}})&\:-i(\sqrt{{\gamma}_{0}{\gamma}_{1}}+\sqrt{{\kappa}_{0}{\kappa}_{1}})&\:{\omega}_{1}-i({\gamma}_{1}+{\kappa}_{1})&\:\cdots\:\\\:\vdots&\:\vdots&\:\vdots&\:\vdots\end{array}\right)\mathrm{.}$$

Here, ${\kappa}_{c}$, ${\kappa}_{0}$, and ${\kappa}_{j}$ ($j\in\left\{1,2,3\right\}$) represent the intrinsic damping rates for the cavity mode, FMR mode, and the $j$^th^ SWR mode, while as ${\gamma}_{c}$, ${\gamma}_{0}$, and ${\gamma}_{j}$ ($j\in\left\{\text{1,2,3}\right\}$) represent the extrinsic damping rates for these modes, respectively. The parameter ${\Theta}\in\left\{0,\pi\right\}$ accounts for the nonreciprocal behaviour induced by the port-dependent phases, where ${\Theta}=0$ is taken if the signal is sent from Port 1, and ${\Theta}=\pi$ is taken if the signal is sent from Port 2^21^. The nonreciprocity induced by the asymmetry of the $\mathbf{h}$ field is phenomenologically captured by adjusting ${\kappa}_{0}$ and ${\gamma}_{0}$.

Assuming that the FMR modes and the SWR modes have the time-dependence of the form ${e}^{-i\omega t}$, we can solve for the observables in terms of the input ${p}_{\text{i}\text{n}}$ –

3 $$\:\begin{array}{c}{\varvec{x}}^{T}=-i\left({\Omega}-\omega I\right)^{-1}{\mathbf{b}}^{T}{p}_{\text{i}\text{n}}\end{array}\mathrm{,}$$

where $I$ is the identity matrix. Finally, we can derive the input-output relationship^31^

4 $$\begin{array}{c}{p}_{\text{o}\text{u}\text{t}}+{p}_{\text{i}\text{n}}=\mathbf{b}\cdot{\varvec{x}}^{T}\end{array}\mathrm{,}$$

The input-output substituting ${\varvec{x}}^{T}=-i\left(\varOmega-\omega I\right){\varvec{b}}^{T}{p}_{\text{i}\text{n}}$ gives

5 $$\begin{array}{c}{p}_{\text{o}\text{u}\text{t}}={p}_{\text{i}\text{n}}\left(1-i\mathbf{b}({\Omega}-\omega I{)}^{-1}{\mathbf{b}}^{T}\right)\end{array}\mathrm{.}$$

For our case, we can compute transmission coefficient as^32^

6 $$\begin{array}{c}{\text{S}}_{21\left(12\right)}=\left(1-i\mathbf{b}(\Omega-\omega I)^{-1}{\mathbf{b}}^{T}\right)\end{array}\mathrm{.}$$

### Simulation of the $\mathbf{h}$ field distribution

The $\mathbf{h}$ field generated by the cross-shaped cavity plays a crucial role in this study, governing the excitation and dynamics of spin precession in the YIG film. The intensity and distribution of the $\mathbf{h}$ field are simulated using CST Studio Suite^33^. Figure 1(e) shows the $\mathbf{h}$ field intensity at the cavity’s resonant frequency with rf excitation from Port 1. The field is stronger near the input side due to asymmetric energy distribution within the cavity. Energy dissipation at the centre reduces the $\mathbf{h}$ field intensity as it propagates toward the cross arms. At the transmission line, adjacent to the input port (left half of the transmission line), the $\mathbf{h}$ field predominantly aligns along the $\:y$-axis and exhibits relatively higher intensity, as indicated by the warmer colours. The $\mathbf{h}$ field gradually transitions from being nearly perpendicular to the $x$-axis at the cavity centre to parallel to the $x$-axis on the two arms. This variation in field direction and intensity highlights the asymmetry in the $\mathbf{h}$ field distribution within the cavity.

The angle between the $\mathbf{h}$ field and the external magnetic field plays a crucial role in the excitation of spin precession. Maximum spin precession occurs when the $\mathbf{h}$ field is perpendicular to the external field, as this maximises the torque on the magnetisation^34^. Conversely, when the fields are parallel, spin precession is inefficient^30^. Therefore, when $\theta=0^\circ$, spins in the two arms of the cross cavity experience strong excitation, while those under the transmission line are minimally excited. At $90^\circ$, this behaviour is reversed, with spins in the cross cavity arms remaining mostly inactive and those under the transmission line experiencing stronger excitation. Consequently, the net torque exerted by the $\mathbf{h}$ field determines the coherent coupling strength, which can be dynamically tuned by adjusting the external field angle^30^.

The $\mathbf{h}$ field directly governs the excitation of the FMR mode, which is most efficient when the spins in the YIG film precess collectively with $\varvec{k}=0$^35,36^. However, its spatially nonuniformity can also excite magnons with finite $\varvec{k}$, introducing spatial variations in spin dynamics^36^. This enables energy transfer from the uniform FMR mode to these magnons, leading to enhanced magnon damping through two-magnon scattering^20,37,38^. Notably, the relative angle between the $\mathbf{h}$ field and the external magnetic field dynamically modulates the strength and spatial distribution of these interactions, offering a means to control FMR dissipation^38^. This variation in angle excites spins in different regions of the YIG film, effectively reconfiguring the inhomogeneities and influencing two-magnon scattering^39^. As a result, the damping rate of the FMR mode is expected to vary with $\theta$. The extrinsic damping rate of the FMR mode is governed by its dissipative coupling with the travelling photon mode. This coupling is influenced by the density of states of the travelling photons, and its contribution can be modulated by $\theta$^34^. This effect plays a key role in determining the strength of the dissipative coupling between the FMR and travelling photon modes^21^.

The $\mathbf{h}$ field distribution excited by Port 2 exhibits $180^\circ$ rotational symmetry relative to the distribution excited by Port 1 (see Supplementary Materials, Fig. S1^44^). This results in different relative angles between the $\mathbf{h}$ field and the external magnetic field when the rf signal is applied from Port 1 versus Port 2. The variation in these angles alters the torque exerted on the magnetisation, leading to differences in spin precession. As a result, the spin dynamics are asymmetric, producing distinct energy dissipation profiles for $\left|{\text{S}}_{21}\right|$ and $\left|{\text{S}}_{12}\right|$, commonly referred to as nonreciprocity^21^. Specifically, the spin excitation and two-magnon scattering process depend on the rf signal direction, which in turn affects the observed transmission characteristics. This nonreciprocity is important for understanding the directional dependence of damping mechanisms and provides insight into how the system can be controlled through the relative orientations of the $\mathbf{h}$ field and external magnetic field.

The intrinsic damping rate of the FMR mode is defined as the total energy dissipation that does not occur through radiative coupling with travelling photons. In our system, the intrinsic damping primarily arises from Gilbert damping and two-magnon scattering, with the latter playing a dominant role. While two-magnon scattering is often classified as an extrinsic mechanism due to its dependence on inhomogeneities, it does not involve photon emission but instead redistributes energy among magnons. As a result, within our framework, two-magnon scattering contributes significantly to the nonradiative damping of the FMR mode, distinguishing it from photon-mediated extrinsic damping. This variation in damping mechanisms plays an important role in modulating the overall system dynamics, further influenced by the asymmetry in the excitation conditions.

### Photon–magnon coupling at $\varvec{\theta}=0^\circ$

**Fig. 2:**
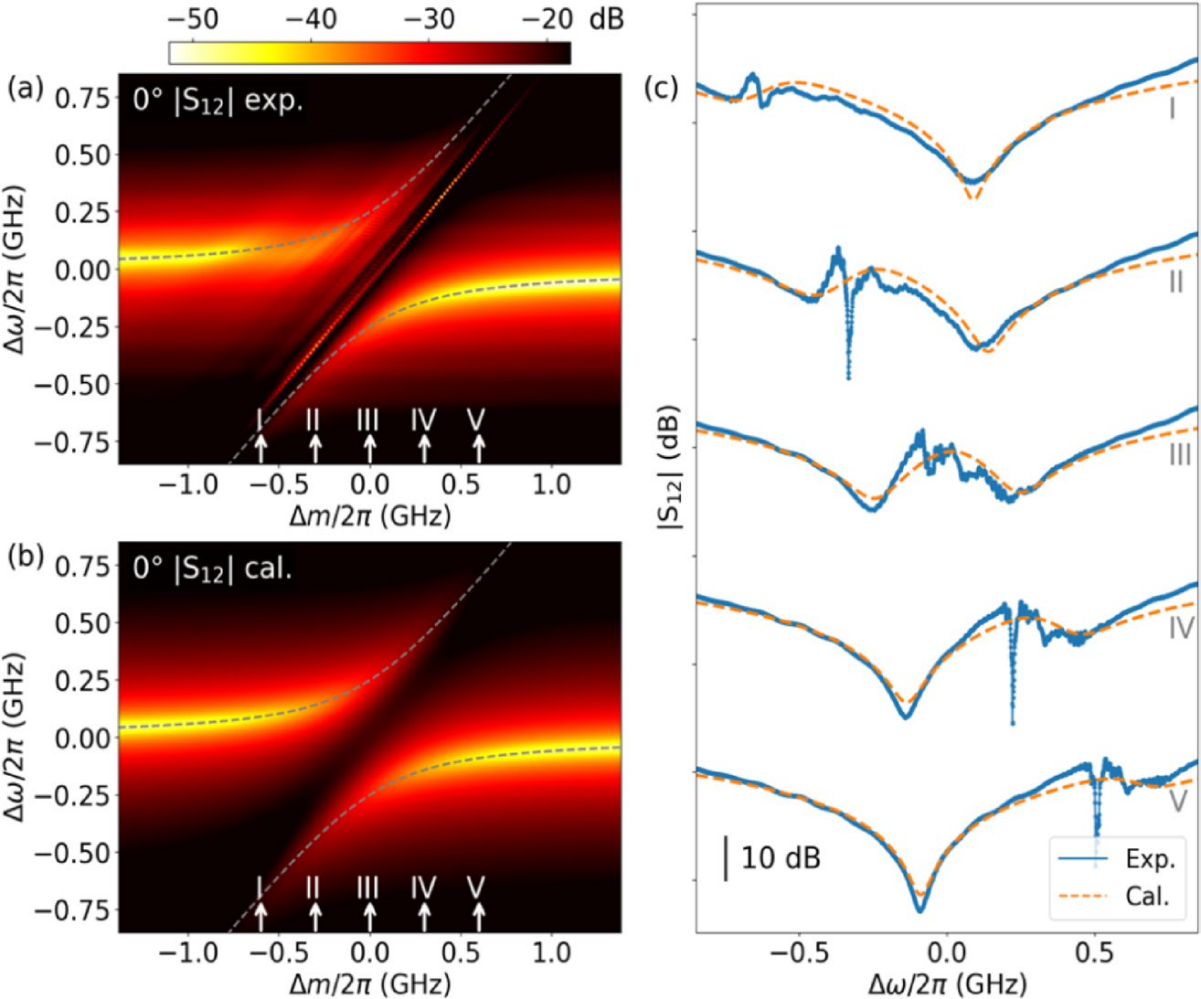
(**a**) Measured and (**b**) calculated $\left|{\text{S}}_{12}\right|$ at $\theta=0^\circ$, plotted as functions of $\varDelta\omega$ and $\varDelta m$, respectively. Dashed lines in (**a**) and (**b**) are the real part of the calculated eigenvalues. (**c**) $\left|{\text{S}}_{12}\right|$ line profiles (solid lines) measured at detuned fields (labelled “I” to “V” in (**a**) and (**b**)), overlaid with calculated curves (dashed lines). A vertical $45\:\text{d}\text{B}$ offset is applied between successive traces for clarity. Lines are a guide to the eye.

Figure 2 presents $\left|{\text{S}}_{12}\right|$ measured at $\theta=0^\circ$. The $\:\left|{\text{S}}_{12}\right|$ intensity is plotted as a function of both the rf frequency detuning ($\varDelta\omega=\omega-{\omega}_{c}$) and field detuning ($\varDelta m={\omega}_{0}-{\omega}_{c}$). Figure 2(c) shows the $\left|{\text{S}}_{12}\right|$ line profiles as a function of rf frequency detuning, measured at the coupling centre (labelled “III”) and at detuned FMR frequencies (labelled “I”, “II”, “IV”, and “V”). A typical photon–magnon coupling is observed, characterised by a cavity mode with a resonance frequency independent of the external magnetic field, an FMR mode whose resonance frequency increases with the field, and an anti-crossing at the coupling centre (${\omega}_{c}={\omega}_{0}$)^2,6,17,40–42^. Within the anti-crossing region, fine lines parallel to the FMR mode correspond to fine spin wave excitations, which are enhanced due to photon–magnon coupling, consistent with previously reports^43^. As in previous studies, these fine spin wave excitations are identified by comparison with their calculated dispersion relations rather than quantitative fitting. Their properties and origin have been systematically characterised in the literature^43^, so our model and fitting analysis is focused on the main hybrid modes relevant to photon and uniform FMR coupling and these fine spin wave excitations are not included in our model. In the spectra, these enhanced fine spin wave excitations appear as sharp peaks at various detuned fields (Fig. 2(c), labelled “II”, “IV”, and “V”), as well as multiple smaller peaks at the coupling centre (labelled “III”).

**Fig. 3:**
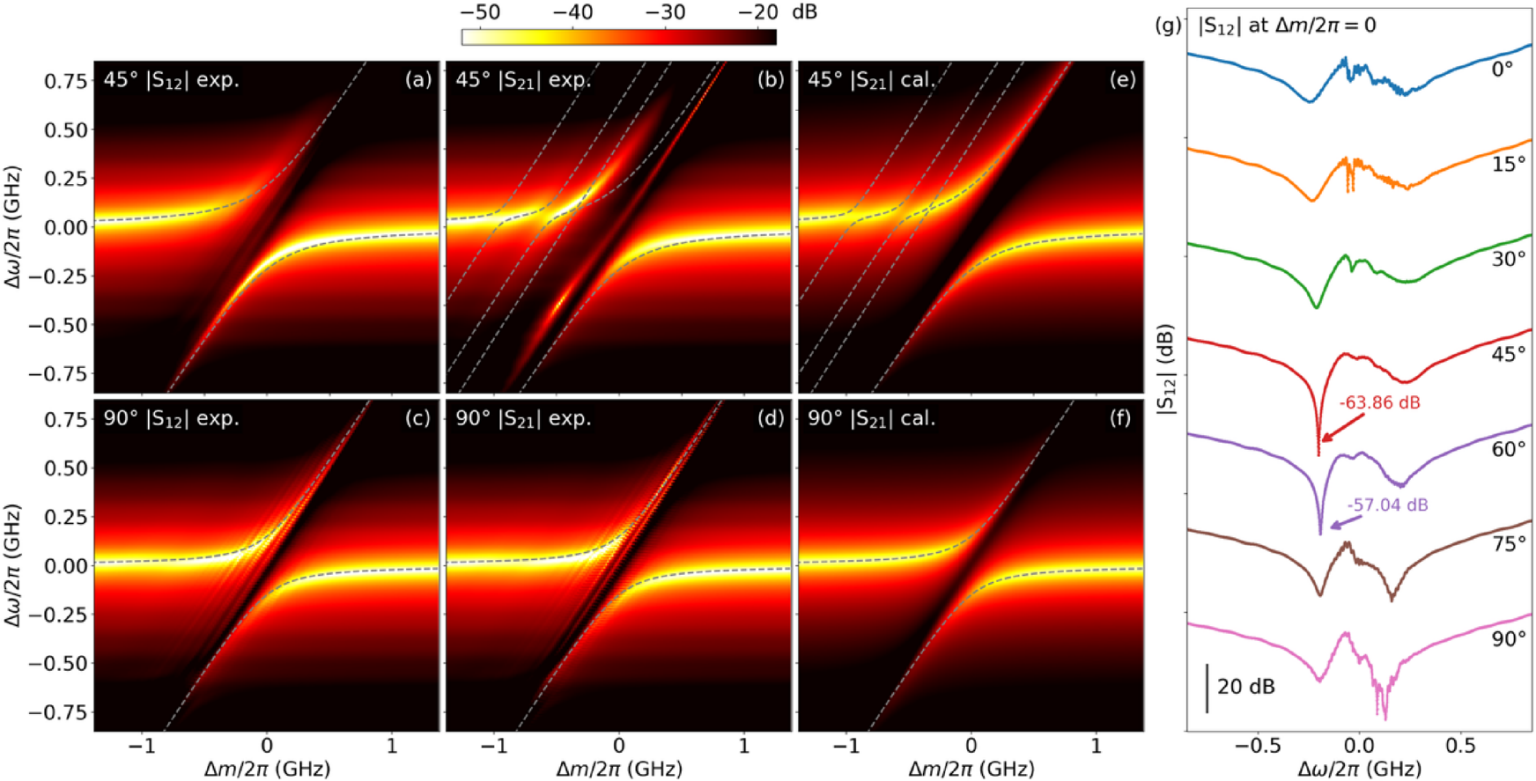
(**a**)–(**d**) Measured $\left|{\text{S}}_{12}\right|$ and $\left|{\text{S}}_{21}\right|$ at $\theta=45^\circ$ and $90^\circ$, plotted as functions of $\varDelta\omega$ and $\varDelta m$, respectively. (**e**), (**f**) Corresponding calculated $\left|{\text{S}}_{21}\right|$ under the same conditions as (**b**) and (**d**), respectively. (**g**) Measured $\left|{\text{S}}_{12}\right|$ at $\varDelta m/2\pi=0$ as a function of $\varDelta\omega$ for $\theta$ from $0^\circ$ to $90^\circ$. A vertical $45\:\text{d}\text{B}$ offset is applied between successive traces for clarity. Lines are a guide to the eye.

As shown in Fig. 2(a), the intensity of the FMR mode rapidly diminishes as the external magnetic field detunes from the coupling centre. The broadened linewidth of the FMR mode in Fig. 3(c) further confirms its high damping rate. This suggests that two-magnon scattering is pronounced when $\theta=0^\circ$, contributing to the broadened linewidth of the FMR mode. Additionally, the FMR mode exhibits a greater intensity when red-detuned compared to blue-detuned, revealing a noticeable asymmetry between the upper and lower branches of the anti-crossing. This asymmetry arises from the dissipative coupling between the travelling photons and the FMR mode^21,34^. The observed asymmetry suggests weak coupling between the FMR mode and the travelling photons.

The cavity-FMR coupling interaction in this case can be described as a direct coupling between the cavity and FMR modes, with a coupling strength of *g*_0_, while both modes also dissipatively couple to the travelling photon modes. Figure 2(b) presents calculated mappings of the transmission parameters as functions of rf frequency detuning and FMR frequency detuning. These calculations use ${g}_{0}/2\pi=240.0$ $\text{M}\text{H}\text{z}$, ${\kappa}_{0}/2\pi=138.2$ $\text{M}\text{H}\text{z}$, and ${\gamma}_{0}/2\pi=12.6$ $\text{M}\text{H}\text{z}$, respectively. Intrinsic damping ${\kappa}_{0}$ here includes the damping owing to the two-magnon scattering, which do not directly couple with travelling photons. Dissipative coupling strength ${{\Gamma}}_{0}$, typically defined as $\sqrt{{\gamma}_{c}{\gamma}_{0}}$, is calculated to be $243.3\:\text{M}\text{H}\text{z}$ in this case. The fitting successfully reproduces the key features observed in the experimental data, validating the proposed model. The calculated line profiles at various ${\Delta}m$ are overlaid on the experimental data in Fig. 2(c), demonstrating excellent agreement.

### Effect of $\theta$ on coupling strength and damping rates

To investigate the dependence of photon–magnon coupling on external magnetic field orientation, we rotate the electromagnets from $0^\circ$ to $90^\circ$. Figure 3(a)–(d) presents measured $\left|{\text{S}}_{12}\right|$ and $\left|{\text{S}}_{21}\right|$ at $45^\circ$ and $90^\circ$, respectively. Compared to $\left|{\text{S}}_{12}\right|$ at $0^\circ$ (Fig. 2(a)), ${g}_{0}$ decreases with $\theta$, as indicated by the fitting results (eigenvalues overlaid in Fig. 3(a)). Specifically, ${g}_{0}/2\pi$ reduces to $215.0$ $\text{M}\text{H}\text{z}$ at $45^\circ$, suggesting that the net torque exerted by the $\mathbf{h}$ field on the magnetisation is weaker than that at $0^\circ$. Furthermore, the asymmetry between the upper and lower branches of $\left|{\text{S}}_{12}\right|$ becomes more pronounced at $45^\circ$. The upper branch diminishes rapidly as the field increases, whereas the lower branch decreases more gradually as the field decreases. Notably, the lower branch exhibits a higher transmission intensity of $-63.86$ $\text{d}\text{B}$ at the coupling centre, compared to $-34.45$ $\text{d}\text{B}$ at $0^\circ$ (Fig. 3(g)). This enhanced transmission and narrower linewidth indicate a reduced FMR damping rate and a weaker two-magnon scattering process. In this work, two-magnon scattering–induced damping is considered part of the intrinsic damping ${\kappa}_{0}$, as it does not directly couple to traveling photons. In contrast, extrinsic damping refers to radiative coupling between magnons and traveling photons. Fitting results reveal a significantly lower ${\kappa}_{0}/2\pi$ of $25.1$ $\text{M}\text{H}\text{z}$ for the FMR mode at $45^\circ$. The increased asymmetry between the upper and lower branches suggests that dissipative coupling between the FMR mode and travelling photons intensifies, while ${\kappa}_{0}$ decreases with $\theta$. The fitted ${\gamma}_{0}/2\pi$ rises to $94.2$ $\text{M}\text{H}\text{z}$ at $45^\circ$. The cavity-FMR coupling at $45^\circ$ is calculated using the same method as for $\left|{\text{S}}_{12}\right|$ at $0^\circ$ (fitting result is shown in Supplementary Materials Fig. S3^44^. The ${g}_{0}/2\pi$ further decreases to $150.0$ $\text{M}\text{H}\text{z}$ at $90^\circ$, representing a $37.5\%$ reduction compared to that at $0^\circ$. The asymmetry between the upper and lower branches nearly vanishes at $90^\circ$, indicating weaker dissipative coupling between the FMR mode and travelling photons relative to $45^\circ$, with a fitted ${\gamma}_{0}/2\pi$ of $6.3$ $\text{M}\text{H}\text{z}$ (shown in Fig. 3(f)). Meanwhile, the FMR linewidth increases, with the fitted ${\kappa}_{0}/2\pi$ reaching $50.3$ $\text{M}\text{H}\text{z}$. The corresponding dissipative coupling strengths ${{\Gamma}}_{0}$ are calculated to be 666.4 MHz at $45^\circ$ and 172.1 MHz at $90^\circ$. Conventionally, level attraction is expected when the dissipative coupling exceeds the coherent coupling. However, in our measurements, we consistently observe level repulsion (anti-crossing), even when ${{\Gamma}}_{0}>{g}_{0}$. We attribute this observation to differences in the treatment of magnon damping in the modelling. In many previous works, the extrinsic magnon damping rate ${\gamma}_{0}$ is often neglected or approximated for model simplicity and qualifiedly fitting. By contrast, our analysis includes the full damping contributions. As a result, the criterion for observing level attraction becomes more restrictive: the cavity extrinsic damping ${\gamma}_{c}$ must be significantly larger than the extrinsic magnon damping rate ${\gamma}_{0}$ (i.e., ${\gamma}_{c}\gg{\gamma}_{0}$)^21^. In our experiments, ${\gamma}_{c}$ and ${\gamma}_{0}$ are of comparable magnitude, so the transition to level attraction is not observed, despite the large dissipative coupling strength.

**Fig. 4:**
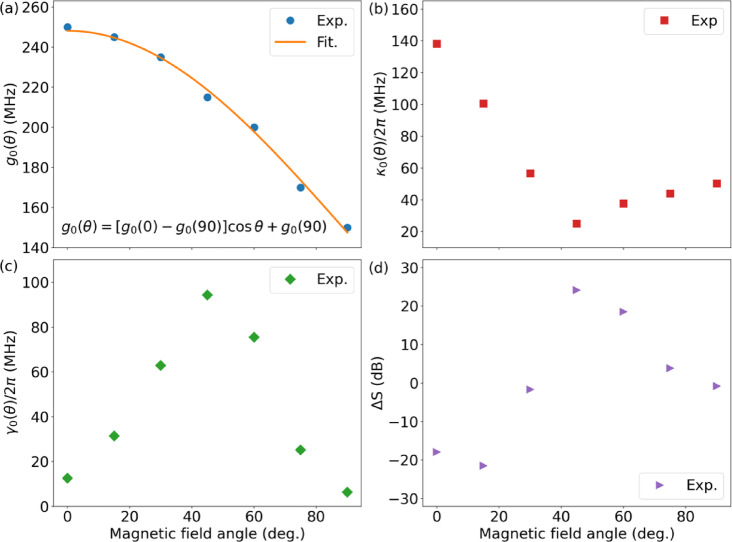
(**a**) Coherent coupling strength *g*_0_ extracted from $\left|{\text{S}}_{12}\right|$ as a function of $\theta$. The solid curve is a fit based on ${g}_{0}\left(\theta\right)=\left[{g}_{0}\left(0\right)-{g}_{0}\left(90\right)\right]\text{cos}\theta+{g}_{0}\left(90\right)$. (**b**) Intrinsic damping rate ${\kappa}_{0}/2\pi$ and (**c**) extrinsic damping rate ${\gamma}_{0}/2\pi$ of the FMR mode excited by Port 2, as functions of $\theta$, respectively. (**d**) Nonreciprocity difference ${\Delta}\text{S}$ as a function of $\theta$.

The coherent coupling strength between the cavity and FMR modes at different field angles ($\left|{\text{S}}_{12}\right|$) is extracted from the model fits and summarised in Fig. 4(a). ${g}_{0}$ decreases continuously with $\theta$, indicating that the net torque exerted by the $\mathbf{h}$ field on the magnetisation weakens as $\theta$ increases. This angular dependence approximately follows a cosine function ${g}_{0}\left(\theta\right)=\left[{g}_{0}\left(0\right)-{g}_{0}\left(90\right)\right]\text{cos}\theta+{g}_{0}\left(90\right)$. The spatial region of spin precession shifts with $\theta$. At $0^\circ$, precession primarily occurs in the cavity arm regions, where the magnetic field is normal to the $\mathbf{h}$ field, maximising the torque exerted on the magnetisation. At $90^\circ$, spin precession is concentrated in the transmission line region, where the magnetic field is perpendicular to the $\mathbf{h}$ field, again maximising the local torque. At intermediate angles, spin precession occurs in both regions. The resulting ${g}_{0}$ reflects the cumulative contribution from spin precession across the entire $\mathbf{h}$ field distribution. Although the $\mathbf{h}$ field is strongest near the transmission line, the cavity arms cover a much larger area. As a result, ${g}_{0}$ is maximised at $0^\circ$ and minimised at $90^\circ$.

The ${\kappa}_{0}/2\pi$ initially decreases sharply from $138.2$ $\text{M}\text{H}\text{z}$ at $0^\circ$ to a minimum of $25.1$ $\text{M}\text{H}\text{z}$ at $45^\circ$ before gradually increasing to $50.3$ $\text{M}\text{H}\text{z}$ at $90^\circ$, representing an overall reduction of over $82\%$ (Fig. 4(b)). This trend is further evident in the line profiles plotted in Fig. 3(g), where the hybridised mode linewidth decreases with increasing field angle up to $45^\circ$, exhibiting a sharp dip, before broadening again as $\theta$ increases. The angular dependence of ${\kappa}_{0}$ indicates that two-magnon scattering is minimised at $45^\circ$ in our setup. In contrast, ${\gamma}_{0}/2\pi$ rises steeply from $12.6$ $\text{M}\text{H}\text{z}$ at $0^\circ$ to a peak of $94.2$ $\text{M}\text{H}\text{z}$ at $45^\circ$, then rapidly declines to $6.3$ $\text{M}\text{H}\text{z}$ at $90^\circ$, marking a reduction of over $90\%$ (Fig. 4(c)). This behaviour suggests that the dissipative coupling between the FMR mode and travelling photon modes is maximised at $45^\circ$. Dissipative coupling strengths ${{\Gamma}}_{0}$ as a function of $\theta$ can be found in Supplementary Materials Fig. S4^44^.

### Nonreciprocity induced by nonuniform rf field

The $\left|{\text{S}}_{21}\right|$ measured at $45^\circ$ exhibits distinct features compared to $\left|{\text{S}}_{12}\right|$, highlighting the nonreciprocal nature of the photon–magnon interaction. While $\left|{\text{S}}_{12}\right|$ captures the fundamental coupling behaviour of the FMR mode, $\left|{\text{S}}_{21}\right|$ reveals additional side couplings at detuned magnetic fields (Fig. 3(b)). These side couplings correspond to strong SWR modes with nonzero $\varvec{k}$ at detuned FMR frequencies of approximately $-0.40$ $\text{G}\text{H}\text{z}$, $-0.65$ $\text{G}\text{H}\text{z}$, and $-1.00$ $\text{G}\text{H}\text{z}$. This nonreciprocity stems from the asymmetry of the $\mathbf{h}$ field in the cavity for forward- and backward-propagating waves. As a result, SWR modes with nonzero $\varvec{k}$ are predominantly excited in $\left|{\text{S}}_{21}\right|$ (the resonant frequency of these SWR modes varies with $\theta$ as shown in Supplementary Materials Fig. S2^44^, while their excitation is suppressed in $\left|{\text{S}}_{12}\right|$. This asymmetry further leads to differences in the ${\kappa}_{0}$ and ${\gamma}_{0}$ of the FMR mode, resulting in variations in the FMR linewidth and the intensity asymmetry between the upper and lower hybridised branches. The nonuniform $\mathbf{h}$ field plays a critical role in selectively driving SWR from one direction, producing an effective nonreciprocal response. As the magnetic field detunes from the resonant frequency, these SWR modes vanish rapidly with broad linewidths, indicating their high intrinsic damping rates. The interaction between these SWR modes and the cavity mode is incorporated into our model, with the calculated spectra shown in Fig. 3(e). The selection of SWR modes is primarily guided by the observed spectra, since their excitation is highly complex due to the nonuniform distribution of the $\mathbf{h}$ field, making it difficult to unambiguously assign a specific wavevector $\varvec{k}$ to each mode. The parameters used for the SWR fitting can be found in the Supplementary Materials Table S1^44^. The calculations accurately reproduce the asymmetric coupling behaviour observed in $\left|{\text{S}}_{21}\right|$. These SWR modes remain observable in $\left|{\text{S}}_{21}\right|$ at $\theta$ ranging from $0^\circ$ to $60^\circ$ (see Supplementary Materials Fig. S2^44^, underscoring the role of the nonuniform $\mathbf{h}$ field and external field angle in driving nonreciprocal magnon-photon coupling.

At $90^\circ$, $\left|{\text{S}}_{21}\right|$ and $\left|{\text{S}}_{12}\right|$ become nearly identical, indicating a transition to reciprocal behaviour. This suggests that $\theta$ plays a crucial role in controlling nonreciprocity in a cavity with a nonuniform $\mathbf{h}$ field. At intermediate angles, such as $45^\circ$, nonreciprocity arises from the asymmetry between the forward- and backward-propagating waves, which is induced by the relative orientation of the $\mathbf{h}$ field and the external magnetic field. The nonuniform $\mathbf{h}$ field, generated by sending signals to Port 1 and Port 2, exhibits $180^\circ$ rotational symmetry. However, for a given $\theta$, the angle between the $\mathbf{h}$ field and the external magnetic field differs for the forward- and backward-propagating waves. This difference in angles leads to variations in the spin precession excitation (including the two-magnon scattering process), which in turn affects the photon–magnon coupling and modulates the reciprocity of the system, transitioning it from nonreciprocal to reciprocal. As $\theta$ further increases to $90^\circ$, this asymmetry diminishes (with the spins precessing most significantly within the transmission line), and the system transitions to reciprocal behaviour, as evidenced by the near-identical $\left|{\text{S}}_{21}\right|$ and $\left|{\text{S}}_{12}\right|$.

To quantify the nonreciprocity of our system, we analyse the nonreciprocity difference, defined as ${\Delta\:}\text{S}=\left|{\text{S}}_{21}\right|-\left|{\text{S}}_{12}\right|$ (with $\left|{\text{S}}_{21}\right|$ and $\left|{\text{S}}_{12}\right|$ in $\text{d}\text{B}$ scale). ${\Delta\:}\text{S}$ is measured at the lower branch of the hybrid mode ($\omega=3.66$ GHz) with zero field detuning (${\Delta}m/2\pi=0$). Figure 4(d) shows ${\Delta}\text{S}$ as a function of $\theta$. At $0^\circ$ and $15^\circ$, $\left|{\text{S}}_{21}\right|$ is stronger than $\left|{\text{S}}_{12}\right|$, resulting in ${\Delta}\text{S}<0$ (around $-20\:\text{d}\text{B}$). At $30^\circ$, ${\Delta}\text{S}$ is nearly zero, while at $45^\circ$, the sign of ${\Delta}\text{S}$becomes positive with a large value of $24\:\text{d}\text{B}$, indicating that $\left|{\text{S}}_{12}\right|$ is now weaker than $\left|{\text{S}}_{21}\right|$. As $\theta$ further increases, ${\Delta}\text{S}$ decreases to nearly zero at $90^\circ$, demonstrating a transition to reciprocal behaviour. These results demonstrate that microwave transmission in our photon–magnon hybrid system can be controlled by adjusting the magnetic field angle, enabling tunable nonreciprocity. The nonreciprocal behaviour in our study contrasts with previous reports where nonreciprocity was attributed to the direction-dependent relative phase between coherent and dissipative magnon–photon couplings^21^. In our work, nonreciprocity arises from two contributions: the first is analogous to the mechanism described in^21^, which is also incorporated into our model. The second and more dominant contribution stems from the differing torques exerted by the nonuniform $\mathbf{h}$ field on the magnetisation for forward- and backward-propagating waves. This torque difference leads to distinct spin precession dynamics and alters two-magnon scattering, thereby affecting the magnon damping rate. This effect depends on the external magnetic field orientation, providing an additional platform for manipulating the nonreciprocity in the photon–magnon coupling system.

## Discussion

The observed variations in coherent coupling strength, intrinsic and extrinsic damping rates of the FMR mode, and nonreciprocity with changing magnetic field orientation underscore the pivotal role of the nonuniform $\mathbf{h}$ field in spin dynamics within the YIG film. These results demonstrate a flexible and experimentally accessible means of controlling critical parameters in photon–magnon hybrid systems. By adjusting the angle between the $\mathbf{h}$ field and the external magnetic field, one can control the torque on the magnetisation, thereby modulating the coherent photon–magnon coupling. Furthermore, the nonuniform $\mathbf{h}$ field enables the excitation of magnons with finite $\varvec{k}$, introducing inhomogeneities within the YIG film that enhance two-magnon scattering. This enhances control over both the FMR linewidth and the dissipative coupling between the FMR and travelling photon modes. Importantly, the spatial asymmetry of the $\mathbf{h}$ field induces direction-dependent excitation condition, leading to nonreciprocal transmission. Unlike conventional nonreciprocity arising from phase differences between counter-propagating modes^21^, the effect here stems from unequal spatial overlaps between the rf field and magnetisation dynamics in the forward and backward directions. This represents a distinct mechanism for achieving nonreciprocal behaviour in cavity magnonics in addition to the phase-difference based nonreciprocity. These findings offer new perspectives on how spatial engineering of the microwave field and magnetic field orientation can be harnessed to tailor photon–magnon interactions. The ability to dynamically control both coherent and dissipative coupling in situ offers promising prospects for reconfigurable nonreciprocal devices, hybrid quantum systems, and spintronic information processing technologies.

We have demonstrated a novel approach for dynamically controlling coherent coupling, magnon damping, and nonreciprocity in a cross-shaped microwave cavity supporting a nonuniform rf field. By rotating the external magnetic field within YIG film, we continuously modulate the torque on magnetisation, allowing precise control over the coherent coupling strength and activation of two-magnon scattering. This scheme enables dynamic and in situ control over magnon dissipation without requiring repositioning of the magnetic sample or changes to the cavity geometry or material properties. Moreover, we realise a distinct form of nonreciprocity arising from spatial asymmetries in the rf field distribution, in addition to phase difference between propagating waves. Our results identify two-magnon scattering as a tunable and reversible control parameter in cavity magnonics and establish a new strategy for engineering coherence, dissipation, and directionality in hybrid light–matter systems. This work provides a foundation for the development of reconfigurable microwave components, nonreciprocal signal processors, and tunable quantum interfaces based on spin dynamics.

## Methods

### YIG film and cross-shaped microwave cavity

The YIG film is $25$ ${\upmu}\text{m}$ thick and measures $4.2$ $\text{m}\text{m}$ $\times$ $3.7$ $\text{m}\text{m}$. The transmission line of the cross-shaped microwave cavity is $0.55$ $\text{m}\text{m}$ wide and $30$ $\text{m}\text{m}$ long. The two orthogonal arms are each $1$ $\text{m}\text{m}$ wide and $8$ $\text{m}\text{m}$ long, totalling $16$ $\text{m}\text{m}$. The cavity is fabricated via photolithography and etching on a Rogers RT/duroid 6010.2LM substrate, which has a dielectric constant of $10.2$.

### Rotatable external magnetic field and transmission spectra measurements

The magnetic field is applied using a pair of rotatable electromagnets, allowing precise control of the in-plane field angle $\theta$ from $0^\circ$ (perpendicular to the transmission line) to $90^\circ$ (parallel). A calibrated vector network analyser (VNA) is used to measure the transmission characteristics of the photon–magnon hybrid system, including $\left|{\text{S}}_{21}\right|$ and $\left|{\text{S}}_{12}\right|$, corresponding to rf signals transmitted from Port 2 to Port 1 and vice versa. The power level is set to $0$ $\text{d}\text{B}\text{m}$, with a frequency sweep from $2$ $\text{G}\text{H}\text{z}$ to $6$ $\text{G}\text{H}\text{z}$ and an intermediate frequency bandwidth of $1$ $\text{k}\text{H}\text{z}$. The measured results are the averages of three scans.

## Supplementary Information

Below is the link to the electronic supplementary material.

Supplementary Material 1

## Data Availability

Data supporting this study’s findings are available from the corresponding author upon reasonable request.
