# Supplementary material for: Reconfigurable control of coherence, dissipation, and nonreciprocity in cavity magnonics
Source: Sci Rep. 2025 Aug 22;15:30893. doi: 10.1038/s41598-025-15983-w (PMC12373738; doi:10.1038/s41598-025-15983-w)
Supplement: Supplementary file 1 — Supplementary Material 1 [file 41598_2025_15983_MOESM1_ESM.docx]

**Supplementary materials for**

**“Reconfigurable control of coherence, dissipation, and nonreciprocity in cavity magnonics”**

**Jintao Shuai**^1^**, Bojong Kim**^1^**, Junyoung Kim**^1^**, Rutvij Bhavsar**^2^**, and Sang-Koog Kim**^1^**^*^**

^1^National Creative Research Initiative Center for Spin Dynamics and Spin-Wave Devices, Nanospinics Laboratory, Department of Materials Science and Engineering, Seoul National University, Seoul 08826, Republic of Korea

^2^School of Electrical Engineering, Korea Advanced Institute of Science and Technology (KAIST), Daejeon 34141, Republic of Korea

^*^Corresponding author: sangkoog@snu.ac.kr

**CST simulations**

Fig. S1(a) shows the $\mathbf{h}$ field intensity at the cavity’s resonant frequency when rf signals are applied from Port 2. Fig. S1 (b) shows the $\mathbf{h}$ field orientation in the YIG region. On the right side of the transmission line, the $\mathbf{h}$ field primarily aligns along the $y$-axis and exhibits higher intensity. Moving towards the cavity centre, the $\mathbf{h}$ field gradually rotates from perpendicular to the $x$-axis to parallel. The $\mathbf{h}$ field intensity and orientation with rf signals applied from Port 2 exhibits $180^{\circ}$ rotational symmetry relative to that from Port 1 (see Fig. 1 in the main text).


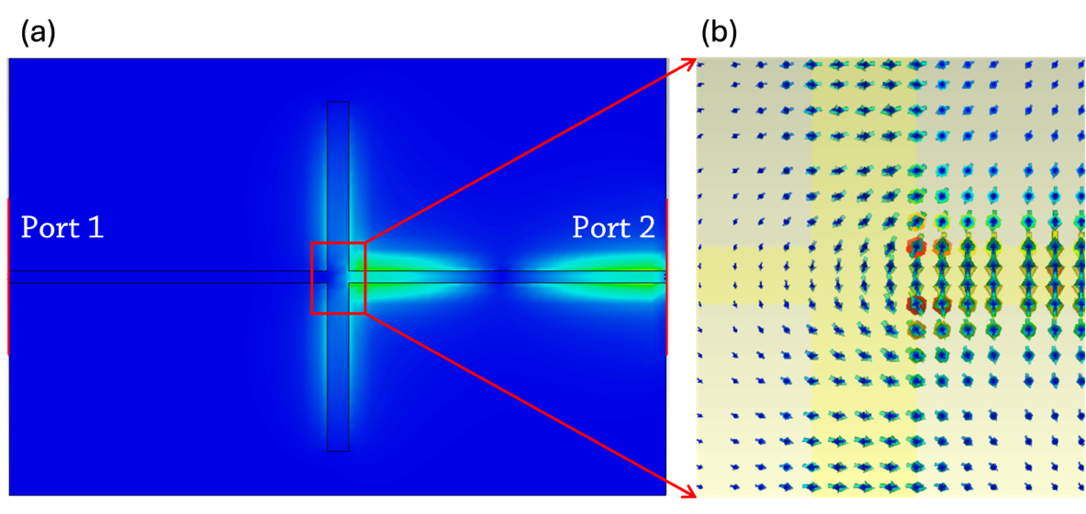


Fig. S1. (a) Heatmap of the simulated $\mathbf{h}$ field intensity in the empty cavity at resonance with the rf signals sent from Port 2. (b) CST simulation of the $\mathbf{h}$ field orientation in the YIG region.

# Measured transmission spectra at different $\boldsymbol{\theta}$

Fig. S2 presents $|S_{21}|$ and $|S_{12}|$ measured as $\theta$ varies from $0^{\circ}$ to $75^{\circ}$, plotted as functions of frequency detuning ($\Delta\omega=\omega-\omega_{c}$) and field detuning $(\Delta m=\omega_{0}-\omega_{c}$). Strong spin wave resonance (SWR) modes are observed only in the upper branch of $|S_{21}|$ from $0^{\circ}$ to $60^{\circ}$ (weakly at $60^{\circ}$), while they are absent in $|S_{12}|$. This behaviour stems from the asymmetry of the $\mathbf{h}$ field, causing different spin precession for forward- and backward-propagating waves. Consequently, the coupling strength between these SWR modes and the cavity modes varies. In $|S_{21}|$, the magnon mode exhibits a narrow linewidth at $0^{\circ}$ and $15^{\circ}$, broadening at $30^{\circ}$ and remaining relatively stable up to $90^{\circ}$. These observations suggest that the two-magnon scattering is minimized around $15^{\circ}$ with a slightly enhanced scattering effect, from $30^{\circ}$ to $90^{\circ}$. By contrast, the magnon linewidth in $|S_{12}|$ decreases with $\theta$ up to $45^{\circ}$ before increasing again towards $90^{\circ}$ (see Fig. 4 in the main text).


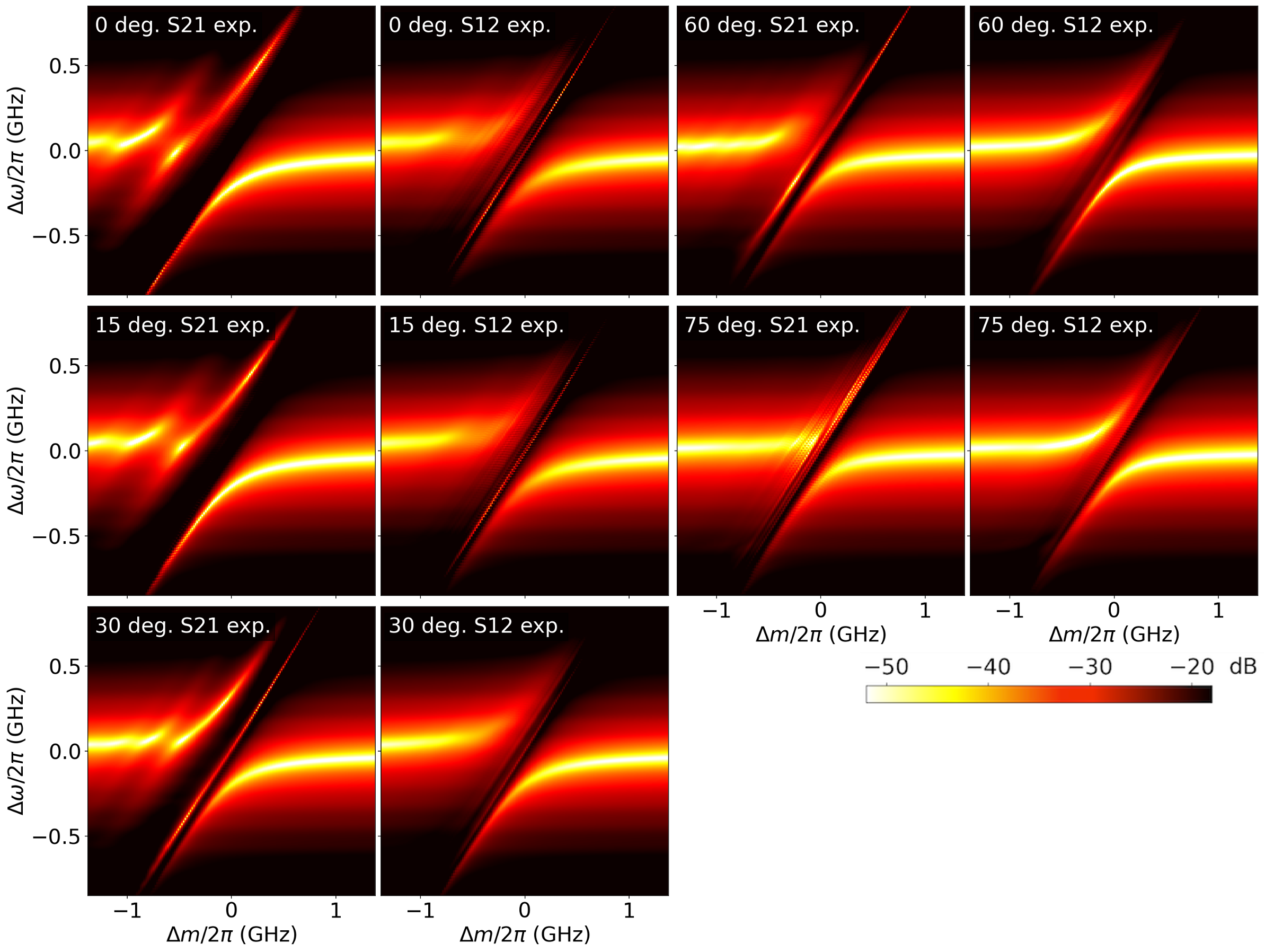


Fig. S2. Measured transmission spectra $|S_{21}|$ and $|S_{12}|$ at an external magnetic field from $0^{\circ}$ to $75^{\circ}$, plotted as functions of $\Delta\omega$and $\Delta m$. The colour bar indicates the transmission in $\mathrm{dB}$.

# Transmission spectra $\boldsymbol{|}\mathbf{S}_{\boldsymbol{12}}\boldsymbol{|}$ at $\boldsymbol{\theta=45}\boldsymbol{^{\circ}}$ and 90$\boldsymbol{^{\circ}}$

Fig. S3 compares the measured and calculated $|S_{12}|$ at $45^{\circ}$ and $90^{\circ}$, plotted as functions of $\Delta\omega$ and $\Delta m$. The fitting captures the coherent coupling between the FMR mode and the cavity mode, while both modes dissipate into traveling photons. The agreement between experiment and calculation is excellent, validating our theoretical approach.


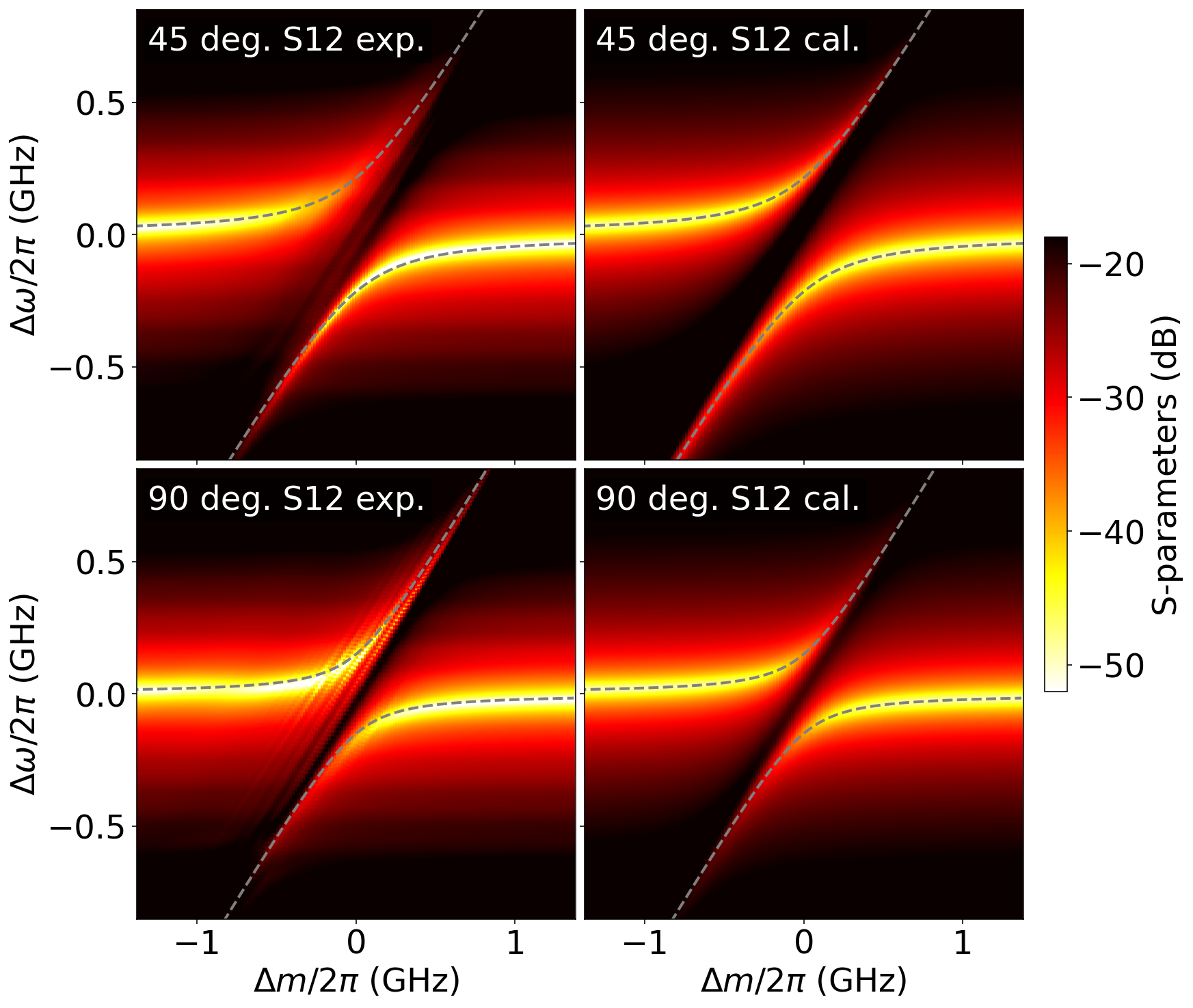


Fig. S3. Measured and calculated $|S_{12}|$ at $45^{\circ}$ and $90^{\circ}$, plotted as functions of $\Delta\omega$ and $\Delta m$. The dashed lines are the real part of the calculated eigenvalues. The colour bar indicates the transmission in $\mathrm{dB}$.

# Fitting parameters used for spin wave resonance modes

The parameters used to fit the spin wave resonance (SWR) modes in $|S_{12}|$ at$45^{\circ}$ in Fig. 3(e) of the main text are summarized in Table S1.

Table S1. Parameters used to fit the spin wave resonance modes in $|S_{12}|$ at$45^{\circ}$.

| Mode $j_{i}$ | $g_{i}$ ($\mathrm{MHz}$) | $\kappa_{i}$ ($\mathrm{MHz}$) | $\gamma_{i}$ ($\mathrm{MHz}$) |
| --- | --- | --- | --- |
| $i=1$ | 10 | 62.8 | 12.6 |
| $i=2$ | 50 | 75.4 | 12.6 |
| $i=3$ | 40 | 125.7 | 12.6 |

# Dissipative coupling strengths between photon mode and FMR modes

The dissipative coupling strength between the cavity photon and the uniform FMR modes is typically defined as $\Gamma_{0}= \sqrt{\gamma_{c}\gamma_{0}}$, where $\gamma_{c}$ and $\gamma_{0}$ are extrinsic damping rates of photon and FMR modes, respectively. Figure S4 presents the extracted $\Gamma_{0}$values of from $|S_{12}|$ measurements, plotted as a function of the magnetic field angle $\theta$.


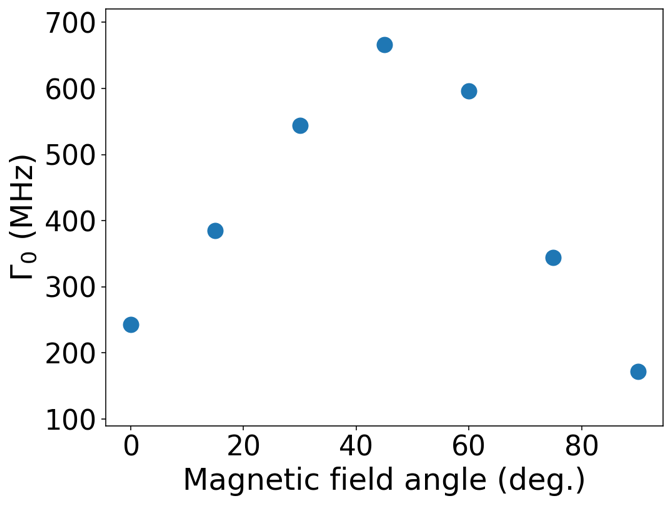


Fig. S4. Dissipative coupling strength, $\Gamma_{0}=\sqrt{\gamma_{c}\gamma_{0}}$, extracted from $|S_{12}|$ as a function of magnetic field angle $\theta$.
